# Nuclear DNA Fragmentation in Boar Spermatozoa: Measurement Methods and Reproductive Performance Implications

**DOI:** 10.3389/fvets.2022.929858

**Published:** 2022-06-30

**Authors:** Raquel Ausejo, Juan Manuel Martínez, Noelia Mendoza, Alfonso Bolarin, M. Teresa Tejedor, Maria Victoria Falceto

**Affiliations:** ^1^Department of Animal Pathology, Obstetrics and Reproduction, Faculty of Veterinary Medicine, University of Zaragoza, Zaragoza, Spain; ^2^Department of Biotechnology R&D, Magapor S.L., Ejea de los Caballeros, Spain; ^3^Department of Research and Development, CiencIAnova Magapor, Zaragoza, Spain; ^4^Department of Anatomy, Embryology and Genetics, CIBERCV, Faculty of Veterinary Medicine, University of Zaragoza, Zaragoza, Spain

**Keywords:** boar, fertility, fragmentation, SCD, sperm DNA fragmentation (sDF), SCSA, TUNEL

## Abstract

The aim of this research was to compare the different techniques to measure sperm nuclear DNA fragmentation (sDF) and to check its relations to boar reproductive value, classical spermiogram parameters, and reproductive results of the doses in sows. Sperm chromatin stability assay (SCSA), terminal deoxynucleotidyl transferase dUTP nick end labeling (TUNEL) assay, and sperm chromatin dispersion test (SCD, Halomax^®^) results were compared, finding a statistically significant correlation only between SCSA and TUNEL results. The fertility direct boar effect (DBE) index, calculated from the whole productive life of the boar, was not correlated (*p* > 0.05) with sDF (measured by any technique). Total or progressive sperm motility was not correlated with sDF, while it found a positive correlation between TUNEL measure and abnormal acrosomes (%) and between SCD measure and total sperm morphological abnormalities (%). No significant correlations were obtained between fertility or prolificacy results and sDF results with the different techniques. However, in the case of total born and SCSA measure, the correlation was close to significance (r partial = −0.095; *p* = 0.066), appointing to a tendency; as SCSA increases, the number of total piglets born decreases. In conclusion, although the different techniques for the sDF seem not to target exactly the same DNA events and the relationship between their values and the reproductive results and the classical spermiogram results is still to be elucidated, the studied sDF techniques may offer extra information that could be useful for the management of AI studs.

## Introduction

The use of artificial insemination (AI) for breeding pigs has been instrumental in facilitating global improvements in fertility, genetics, labor, and herd health [reviewed by (1)]. Nowadays, a single boar inseminates about 2000 sows yearly and the aim of 6000 sows per boar in the near future is a feasible expectation [reviewed by (2)]. Today, each boar ejaculate is managed for the production of 20 to 40 traditional AI doses containing 2,000 to 3,000 million total motile sperm in 75 to 100 mL of extender or 40 to 60 doses with 1,000 to 2,000 million total sperm in similar or reduced volumes for use in post-cervical or intrauterine AI. However, efforts are aimed at methods to continue to lower the number of sperm in an AI dose to produce a higher number of doses per boar reducing the costs and spreading the genetic advantages on to more production farms. To achieve this goal, boar sperm quality has to be unquestionable in order to guarantee pregnancy and high prolificacies even with a lower number of sperm cells.

The classical approach for evaluating boar sperm quality relies on sperm motility and morphology evaluated by microscopy (3). However, there are some semen traits affecting fertility not identified in the conventional spermiogram occurring at a molecular level as is the case of sperm nuclear DNA fragmentation (sDF) that could be used as fertility biomarkers (2, 4). It consists of the single- or double-strand breaks in the nuclear DNA of the spermatozoon resulting in a potential loss/alteration of genetic information. Damaged sperm chromatin may impair the capability of the spermatozoa to fertilize an oocyte, decrease insemination success, cause abortion or fetal abnormalities, and even reduce offspring vitality (5).

The present research deals with three different techniques to measure sDF:

The **sperm chromatin stability assay (SCSA)** is the most widespread test. It is a simple test that assesses sperm nuclear chromatin status by flow cytometry using acridine orange (AO) fluorochrome (6). This stain intercalates in the DNA fluorescing green when associated with double-stranded DNA (native) and red when associated with single-stranded DNA (ssDNA, denatured). The denaturation step induces the formation of ssDNA from breakages; therefore, each sperm head yields a mixture of green and red fluorescence when interrogated with a 488-nm laser, depending on the DNA fragmentation (number of nicks) and the susceptibility of chromatin to denaturation. The DNA fragmentation index (DFI) refers to the percentage of spermatozoa in the region of the flow cytometry histogram that is designated according to red/green fluorescence ratio (7).

Another flow cytometry DNA assay is the **TUNEL (terminal deoxynucleotidyl transferase-mediated deoxyuridine triphosphate-nick end labeling) assay**. This technique has been widely used because it measures a definitive end point (the presence of free 3′ hydroxyl groups), is not technically challenging, and, unlike the acridine orange-based SCSA assay, does not require dedicated flow cytometer maintenance (8, 9). However, this assay is more expensive to perform than the SCSA. A particular feature of this assay is that it depends upon the ability of a protein, terminal transferase, to access DNA strand breaks and catalyze the insertion of labeled bases. While this might not be a problem for somatic cells, the highly specialized, compacted nature of sperm chromatin seriously restricts such access. TUNEL demonstrated a good relationship with SCSA and with fertility in humans (10) and bulls (11), but correlations in pigs have not been yet elucidated.

The other technique tested was the **SCD (sperm chromatin dispersion)** test, a recently developed technique for assessing sperm chromatin, which is relatively simple and inexpensive. It is based on the inclusion of spermatozoa in a gel matrix, applying a high-salt low-pH treatment (including reducing agents to break disulfhydryl bonds in the chromatin). After this treatment, the sample is analyzed by bright-field or fluorescence microscopy, obtaining the percentage of sperm heads with a halo. Depending on the commercial kit used, the halos indicate either good or bad condition of the sperm DNA (12, 13). Halomax^®^ (Halotech DNA SL) is a commercial trademark of an SCD test for veterinary use to measure sDF for a specific animal species having a particular kit for boar. The test is relatively easy to perform and does not require expensive equipment, but can be very time-consuming if analyzing many samples because it is recommended to count at least 500 spermatozoa each time. It is not sure whether SCD has a relationship to fertility in humans (14) although some studies indicate that a relationship indeed may exist and that it may have a performance comparable to SCSA or TUNEL (9). However, its high variability may hamper its diagnostic and prognostic use (15, 16). In particular, this test has yet to be compared to the other techniques in pigs.

Considering the great number of factors that can impair the nuclear DNA integrity of the spermatozoa, either during spermatogenesis (genetics, health, and environment) or afterward (contaminations, oxidative damage, manipulation), an effective and affordable test to assess the sperm DNA integrity should be considered as an extra requirement for a complete spermiogram (17).

The aim of this research was to compare three different techniques to measure sperm nuclear DNA fragmentation (SCSA, TUNEL, and SCD) and to study its correlation with the conventional spermiogram parameters and the fertility and prolificacy results of sows inseminated with these ejaculates. The final objective was to figure out whether sDF could be used as a fertility biomarker.

## Materials and Methods

The study includes approved methods and standard operating procedures for boar semen processing. The AI studs in this study complied with the Council Directive, 2008/120/EC outlining minimum standards for the protection of pigs and Directive, 2010/63/EU of the European Parliament and the Council of 22 September 2010, on the protection of animals used for scientific purposes.

This study consists of two experiments:

In **experiment 1**, we compared results from the aforementioned three sDF measurement techniques on data collected from a large database (5,424 ejaculates) and studied the potential relationships between these measures and the fertility/prolificacy direct boar effect (DBE) score of each individual male, as a summary of his productive life.

In **experiment 2**, we analyzed the relationship between sDF measures in one ejaculate of each 58 individual boars and both basic spermiogram characteristics and the fertility results of inseminations of the evaluated ejaculates in commercial farms in terms of fertility and prolificacy of the sows mated.

### Boars and AI Studs Involved

#### Barns and Animals

The data were collected from four different commercial AI studs of three different boar semen production companies located in Spain, which were selected based on the production procedures, housing systems, and boar management practices. The AI studs had between 80 and 300 boars. Boars were chosen randomly at the beginning of the study. When applicable, the fertility records of the donors were gently offered by AIM Ibérica (Topigs Norsvin Spain, Spain). In total, different data from 5,424 ejaculates collected along all the seasons from 1,900 different boars were registered in the database between 2018 and 2020. The distribution (mean ± SD) of the age of the boars at collection was 18.5 ± 9 months, and the breeds were mostly commercial lines of Duroc, Landrace, Large White, and Pietrain. Boars were allocated in individual crates of at least 6 m^2^. All facilities had water available *ad libitum*, and animals were fed 1–2 times per day a total of 2.5–3.5 kg with a commercial boar feed (9.2 MJ/kg ME, 15.5% protein, 4.5% fiber, 12,500 IU/kg vit A, and 150 IU/kg vit E). Although weather conditions differed among the seasons, light exposure was controlled in all the barns, with more than 150 lux at eye level during 14–16 h per day and limited contact with natural light. Every facility also maintained a controlled temperature inside, ranging from 11°C (coldest, in winter) to 28°C (hottest, in summer).

#### Collection and Semen Sampling

In all cases, ejaculate collection was performed by experienced technicians in a separate collection room. Double glove technique and hygienic measures were taken with previous dry cleaning of the preputium and penis before collection. Ejaculate collection was performed with semi-automatic devices in all cases. All the ejaculates were collected, including sperm-rich and post-sperm-rich fractions. Bacteriology was checked for each ejaculate in blood agar plates (37°C during 48 h) with always <300 CFU/ml (data not shown).

### Classical Spermiogram Analysis

#### Sperm Concentration and Morphology

Sperm concentration was assessed with a spectrophotometer (Magapor S.L., Spain) in duplicate.

Sperm morphology was assessed on a slide extension using eosin-nigrosin staining (18). Stained sperm were individually evaluated by experienced staff using a phase-contrast microscope at 200 magnification. A total of 100 sperm cells were checked per each ejaculate. Sperms with abnormal morphology were recorded. An abnormal sperm cell included one or several of the following events: defective acrosome, abnormal heads, tail or mid-piece defects, and proximal or distal cytoplasmic droplets. These abnormalities were registered in the order cited, and if a sperm cell has more than one abnormality, just the first one in the hierarchy order is registered.

#### Motility Analysis by CASA

A 3.5 μL drop was pipetted into a Magapor counting chamber (20-μm depth; Magapor S.L.) and evaluated with a phase-contrast microscope (Motic BA310 series with a warmed stage at 37°C; 10 × negative contrast optics). Image sequences were acquired from at least three independent fields (at least 200 motile spermatozoa per sample, except in those with extremely low motility). Image sequences were analyzed with Magavision Sci software (Magapor SL), for total and progressive motility.

### Sperm Nuclear DNA Fragmentation Technique

Semen samples were collected at the boar collection centers for sDF. Ejaculates were taken to a production facility, and samples were pre-diluted with TNA buffer (0.01 M Tris-HCl, 0.15 M NaCl, and 1 mM disodium EDTA; pH 7.4) within 5 min after collection, aiming for a concentration of 20 million sperm cells per ml. Diluted samples were stored immediately at −20°C and then sent to the research laboratory in Polystyrene boxes with controlled freezing temperature for the following 24 h. After arrival, samples were stored at −80°C until further processing.

sDF was assessed by the three different techniques described below:

-**SCSA** as described by Evenson (7). For analysis, an aliquot of 200 μl of each sample was thawed and treated with 400 μl of a solution containing 0.1% Triton X-100, 0.15 mol/L NaCl, and 0.08 mol/L HCl pH 1.2. Then, 1.2 ml of staining buffer (6 μg/ml acridine orange, 100 mmol/L citric acid, 200 mmol/L Na_2_HPO_4_, 1 mmol/L disodium EDTA, 0.15 mol/L NaCl, and pH 6.0) was mixed with the semen sample after 30 s. The resulting sample was analyzed by flow cytometry (FACS, Becton Dickinson, Madrid, Spain) using the software CellQuest v3 (Becton Dickinson), counting more than 2,000 events (aiming at 5,000). Histogram plots (total sperm cells vs DFI) and DFI readings were calculated for each sperm cell. DFI is used to refer to the ratio between the red fluorescence and the total fluorescence (red + green) of individual spermatozoa (6). Spermatozoa that individually surpassed the values were classified as total DFI (tDFI).

**-TUNEL**: The presence of apoptosis-like DNA strand breaks was evaluated by the TUNEL assay using an *in situ* Cell Death Detection Kit with fluorescein isothiocyanate (FITC)-labeled dUTP (Roche, Mannheim, Germany). Sperm samples were fixed with paraformaldehyde in PBS (Phosphate-buffered saline) at RT (room temperature) for 1 h. After two washes with PBS at 600 × g for 10 min at RT, the samples were permeabilized with 0.1% Triton X-100 in 0.1% sodium citrate for 2 min at 4°C. The reaction was performed by incubating the obtained pellet with 50 μl of labeling solution (TdT enzyme and dUTP: terminal deoxynucleotidyl transferase and deoxyuridine triphosphate) for 1 h at 37°C in the dark. Negative control was prepared by suppressing TdT enzyme from the reaction mixture. After, two washes with PBS were performed to stop the reaction, and flow cytometry analysis was carried out. Positive controls were simultaneously prepared by additional treatment with 10 IU DNase I for 10 min at 15–25°C before the elongation reaction.

A BD Accuri™ C6 with BD software (Becton Dickinson, Madrid, Spain) was used for all the measurements, and at least 40,000 events were counted in every experiment. The sperm population was gated for further analysis based on its specific forward (FS) and side scatter (SS) properties; other non-sperm events were excluded. TUNEL positive spermatozoa were evaluated in filter FL1 (533/30) and considered DNA-damaged.

**-SCD:** Sperm chromatin dispersion was evaluated immediately after thawing using the Sperm-Sus-Halomax^®^ kit (Halotech DNA SL, Madrid, Spain) following a procedure described by Alkmin et al. (19). A minimum of 300 sperm were microscopically evaluated for each sperm sample, and sperm exhibiting a large scattered halo around the head were considered to have fragmented nuclear DNA.

The three different techniques to measure sDF were compared. There were 54 data from 53 boars registered for TUNEL assay, 1,099 data from 672 boars registered for SCD analyses, and 5,424 data from 1,900 boars for SCSA (tDFI) measurements.

### Relationship Between sDF and the Direct Boar Effect (DBE)

At Topigs Norsvin Research Center B. V. (Beuningen, the Netherlands), a breeding database (Pigbase) is available containing fertility records from purebred and crossbred swine farms that are recording fertility data. Sow fertility data are corrected for farm- and sow-related factors (e.g., parity, genetic line sow, farm, season, first or re-mating, purebred/crossbred, number of inseminations, and age semen), and the remaining variation is the direct boar effect (DBE).

All the males in the DNA fragmentation study have scores according to their reproductive parameters. DBE indexes are calculated from all the available reproductive outputs of the male to the date comparing the value of the boar to the average of his population. These indexes are calculated from best linear unbiased prediction (BLUP) models. Boars that have positive DBE have mean results above the arithmetic average of the population, while negative values (below zero) mean poorer reproductive outcomes (below population average). DBE for prolificacy is expressed as the number of piglets, while DBE for fertility is expressed as a percentage.

### Reproductive Data From Inseminated Sows

After a comparison of the different techniques and comparison of the sperm DNA fragmentation to the DBE of each male, sDF indexes of 58 specific boars were compared to the characteristics of the basic spermiogram and to the current results of their inseminations in commercial farms in terms of fertility and prolificacy of the sows mated. Post-cervical doses of 45 mL were prepared to aim for a concentration of 40 million sperm cells per mL diluted in Biosem (Magapor SL). Sows were inseminated post-cervical after spontaneous ovulation with the doses stored for no more than 48 h at 16°C.

The studied 58 boars belonged to six pig breeds: Duroc (10), Landrace (29), Large White (16), Pietrain (1), and York (2). The ejaculates of these boars followed up the doses to thirty-eight commercial sow farms to inseminate 502 sows. Farm size was variable from 500 up to 2,800 sows, and the inseminations were performed in the months of March, April, and May 2021. Mixed parity (average 1.92 ± 1.75; from one to eight) Large White x Landrace sows were used. Farms were located in different areas of Spain.

Individual reproductive data from the inseminated sows were registered: fertility, total born piglets, live-born, and stillborn piglets. The values for fertility were coded as 0 (no farrowing) and 1 (farrowing) and the rest of the values as the number of piglets.

### Statistical Analyses

Data were analyzed by IBM SPSS Statistics 26.0 software package (SPSS, Chicago, IL, USA). All quantitative variables studied were tested for normality distribution by the Shapiro–Wilk test; fragmentation variables were summarized as median and interquartile intervals [IQR]. A non-parametric Spearman correlation (Rho) was run to assess the strength of association between two fragmentation variables. Partial correlation, adjusting for sow's cycle, was applied when studying the fragmentation effect on total born piglets and stillborn piglets. Regression models were applied to predict the evolution of a dependent variable as a function of one or more independent variables. Relationships of fragmentation and fertility were studied by binomial logistic regression. *P*-values <0.050 were considered statistically significant.

## Results

### Comparison Among the Sperm Nuclear DNA Fragmentation Indexes Obtained With the Different Techniques

SCSA (tDFI) data ranged from 0.00 to 94.5% (median [IQR]: 0.930% [1.600%]); TUNEL ranged from 0.1 to 74.2% (median [IQR]: 1.650 % [4.9 %]), and SCD ranged from 0.0 to 50% (median [IQR]: 1.330% [1.663%]). The data significantly departed from normal distribution (Shapiro–Wilk test, *p* < 0.05). Logarithmic transformation of tDFI values did not improve these departures.

The Spearman correlation coefficients were calculated for all the relations between the values obtained with the different techniques (TUNEL, SCD, and SCSA). A positive highly significant and low Spearman correlation (Rho = 0.383; *p* = 0.004; *n* = 54) was observed between TUNEL and SCSA (tDFI) values. Linear regression of SCSA (tDFI) on TUNEL explains 10.4% of SCSA (tDFI) variation. No statistically significant correlations between TUNEL and SCD (*p* = 0.444; *n* = 18) or SCSA and SCD (*p* = 0.198; *n* = 815) were found.

### Relationship Between sDF and DBE Score for Fertility and for Total Born and Stillborn Piglets of Each Male

For the correlation study of TUNEL analysis and DBE (fertility, total born, and stillborn piglets), 440, 331, and 331 data pairs, respectively, were used. For tDFI (SCSA), there were available 1,299 data pairs from 287 boars. From some boars, there was only one data pair, but, however, in other cases, there were several values of tDFI and only one individual DBE value for total born and only one individual DBE value for fertility. Therefore, data pairs for correlation study were created from the average tDFI value per individual and the unique values of DBE for fertility and prolificacy, respectively. Correlation studies for SCSA with fertility included 502 data pairs while for SCSA with total and stillborn piglets included 371 data pairs. Finally, for SCD analyses, there were 252 data pairs for correlation with fertility and 182 data pairs for correlations with total born and stillborn piglets.

No statistically significant correlations (*p* > 0.05) were found in any case for sDF measure (TUNEL, SCSA, or SCD) with DBE for fertility or total born and stillborn piglets.

### Relationship Between sDF and Characteristics of the Spermiogram

The second part of the research studied the correlation between sDF measurements from the different techniques (TUNEL, SCSA, and SCD) and several characteristics of the particular ejaculate where sDF was measured. For this study, ejaculates from 58 boars were used.

Correlations between sDF measures (SCSA, TUNEL, and SCD) and sperm motility were studied. No statistically significant correlations between any sDF measurements SCSA, TUNEL, or SCD and total or progressive motility were found.

Similarly, correlations between sDF measures (SCSA, TUNEL, and SCD) and several morphological abnormalities were studied. These anomalies were taken into account individually, that is, acrosomal defects, abnormal heads, loose heads, abnormal tail, bent tail, and proximal and distal droplets. A significant positive and low correlation was found between TUNEL and abnormal acrosome morphology detected by eosin stain (Spearman's Rho = 0.384). No significant correlations between any sDF measures and the rest of morphological abnormalities were found (*p* > 0.05).

**Figure 1:**
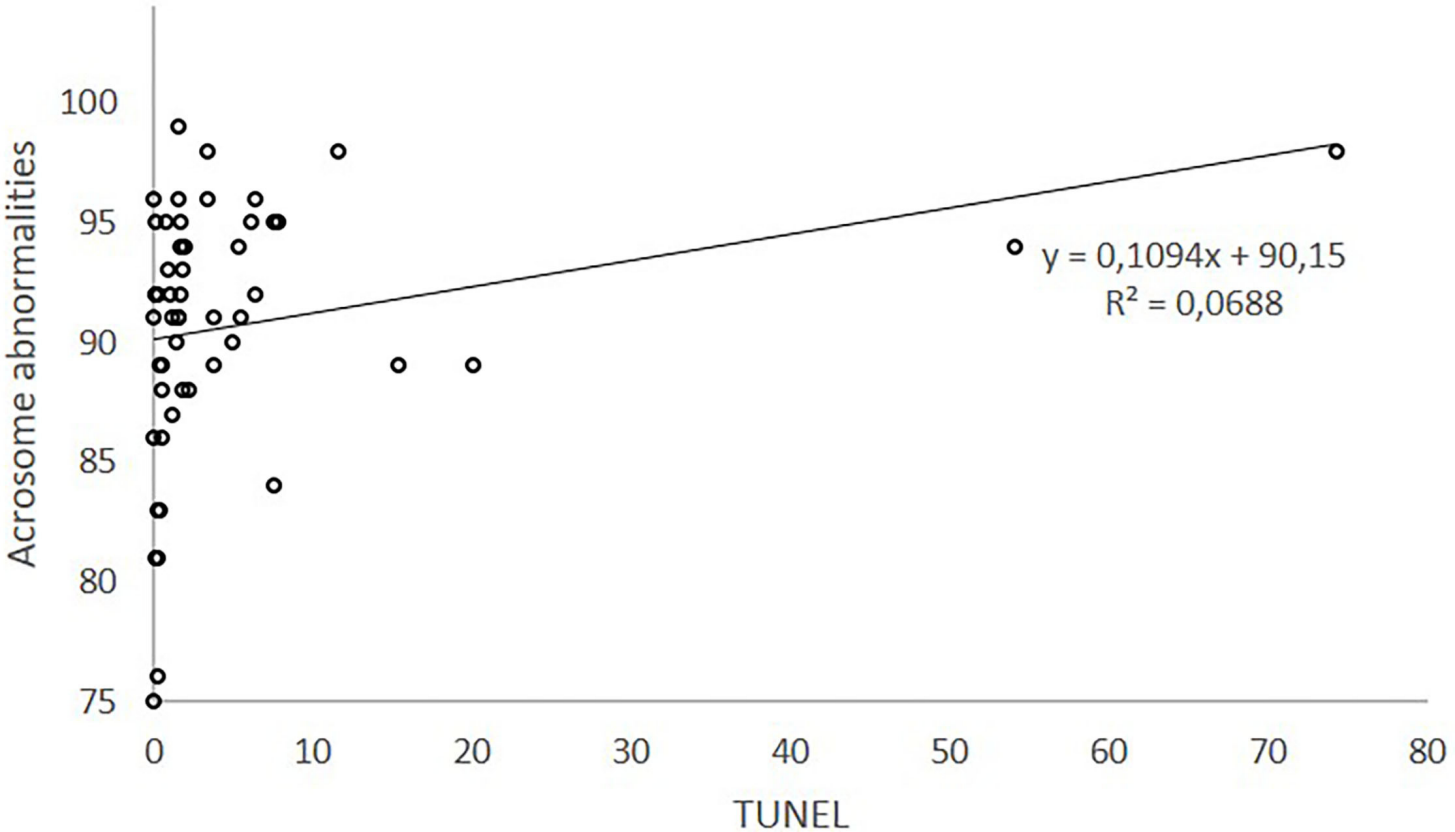
Relationship between acrosome defects and sDF measured by TUNEL.

Linear regression of acrosome abnormalities (%) on TUNEL explained for 6.9% of acrosome abnormalities. Figure 1 plots the relationship between acrosome abnormalities and TUNEL. The equation obtained (y = 0.1094^*^x + 90.15) explains the 6.9% of the variability of acrosome variation. According to this equation, acrosome abnormalities (%) would increase 0.10 units per increased unit of TUNEL measurement.

On the contrary, when morphological abnormalities were globally considered (total abnormalities, %), no statistically significant correlations between SCSA or TUNEL and abnormal sperm were found (*p* > 0.05). However, a statistically significant positive and medium correlation between SCD and total abnormalities was found (Spearman's Rho = 0.475; *p* = 0.030).

The equation obtained (y = 8.7675^*^x + 21.416) explains 34.2% of the variability of total abnormalities. According to this equation, total morphological defects (%) would increase 8.7675 units per increased unit of SCD measurement. Linear regression of total abnormalities (%) on SCD explains 34.2% of total abnormalities.

### Relationship of sDF and Reproductive Outputs (Fertility and Prolificacy) of Sows Inseminated With the Ejaculates

Individual reproductive data from 502 inseminated sows were compared to sDF analyses from 58 ejaculates. There are 502 fertility outcomes, 374 valid data of total born, live-born, and stillborn piglets, 440 TUNEL data, 252 SCD data, and 502 SCSA data.

The binomial logistic regression technique was applied to determine whether there was any relationship between fertility and the different sDF measurement techniques and cycles. No significant effect of the cycle or sDF technique was detected on the final reproductive result (*p* > 0.05).

Linear regression analysis only showed a highly significant effect of sow's cycle on total born piglets (*p* < 0.01): As the sow cycle increases, the number of total piglets born increases. No significant effect of sDF was found (*p* > 0.05). In addition to this regression study, statistical analysis was performed to figure out partial correlations between total born piglets and sDF, adjusting for the sow's cycle. Table 1 shows the results. No significant correlations were obtained in any case (*p* > 0.05). However, in the case of total born piglets and SCSA measure, the correlation was close to significance (r partial = −0.095; *p* = 0.066), appointing to a negative tendency; as SCSA measure increases, the number of total born piglets decreases.

**Table 1:** Partial correlations between sDF (SCSA, TUNEL, and SCD) and total born piglets, adjusting for the sow's cycle.

**Variable**	**Correlation coefficient r**	**df**	***P*-value**
SCSA (tDFI)	−0.095	371	0.066
TUNEL	−0.057	331	0.296
SCD (Halomax^®^)	0.021	182	0.772

Likewise, statistical analysis was performed to figure out partial correlations for percentage of stillborn piglets, adjusting for the sow's cycle. No significant correlations were obtained in any case (*p* > 0.05).

## Discussion

### Comparison Among the sDF Indexes Obtained With the Different Techniques

While SCSA and TUNEL are techniques based on flow cytometry, SCD is analyzed by bright-field or fluorescence microscopy. The resolution of flow cytometry is much higher counting 5,000 events, while in SCD is recommended to count at least 300 spermatozoa but can be very time-consuming if manually analyzing many samples and the operator could suffer visual fatigue missing results. The SCD is a “simple” method in kit form. Unlike all the other tests, it measures the absence of damage rather than the damaged DNA in sperm. In our research, no statistically significant correlations between TUNEL and SCD or SCSA and SCD were found. SCD technique is a low-resolution technique, that counts, as applied in the present research, few sperms per sample, with low repeatability (19) and thus not comparable to SCSA (7). Martínez-Pastor et al. (20) compared SCSA and SCD data to assess the chromatin status of three individual samples of cryopreserved bull semen, analyzed after thawing and after 6 h at 37°C with and without oxidative stress. While SCD could not discriminate between samples with and without oxidizing treatment, SCSA (%DFI) showed a high discriminating ability. Moreover, the repeatability coefficient indicated lower repeatability for SCD when compared to SCSA.

**Figure 2:**
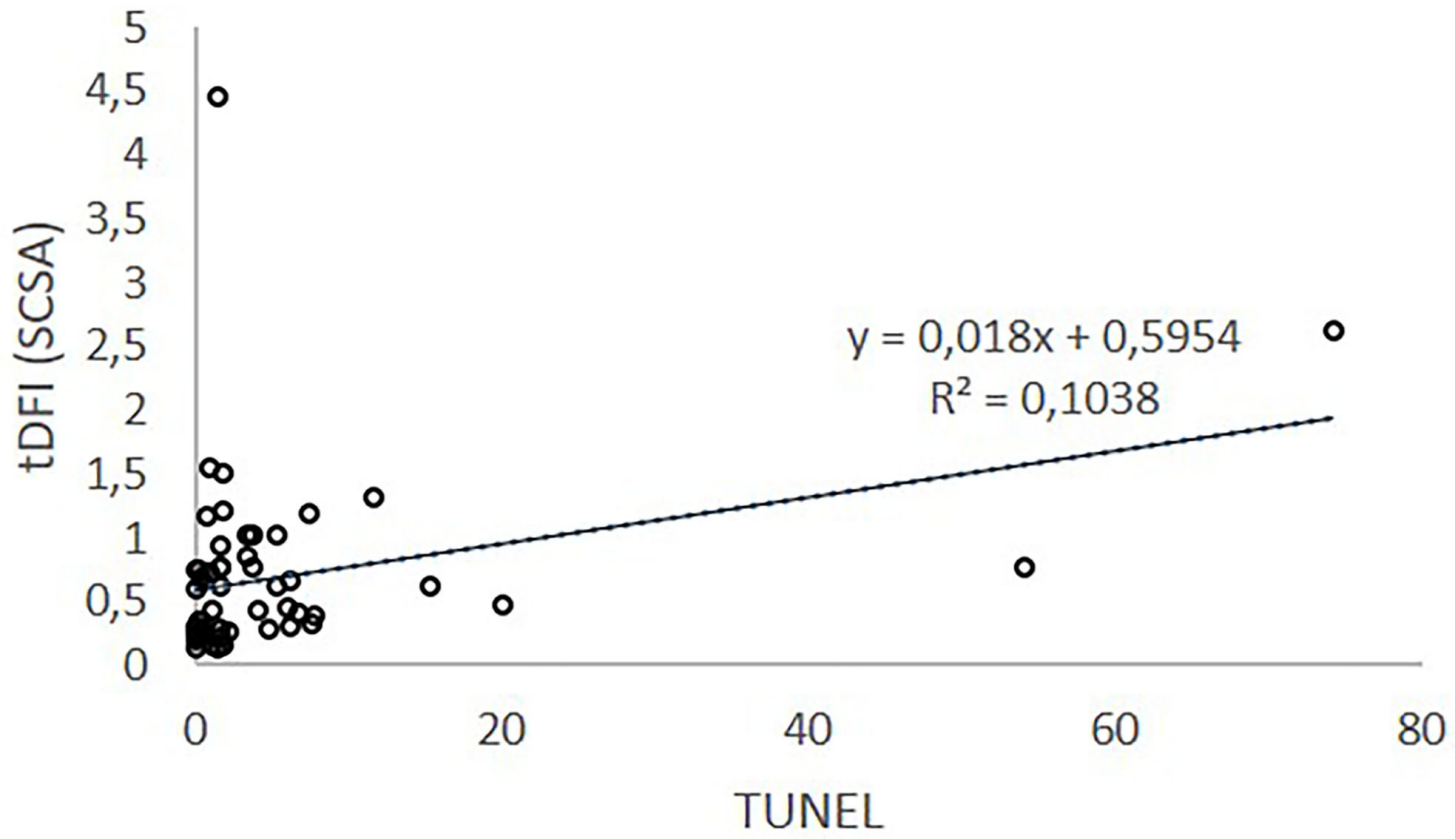
Relationship between sDF index measured by SCSA and TUNEL.

On the contrary, although TUNEL and SCSA methodology to detect DNA damage or weakness are different (20), their results can be considered equivalent (7). According to our results, a positive highly significant correlation was found between SCSA and TUNEL measures. The SCSA pioneer author (7) illustrated data of % DFI (TUNEL) vs. % DFI (SCSA) from human, bull, ram, and stallion. In his research, high-level correlations were found suggesting that SCSA and TUNEL are measuring the same DNA defect. However, SCSA test is supposed to have greater sensitivity for measuring DNA strand breaks throughout the entire chromatin complex in contrast to the TUNEL test. While the TUNEL test requires the TdT enzyme to add dUTP to broken DNA ends and access to a very compacted sperm chromatin, the SCSA requires only the entry of the very small AO molecule that likely detects lesions in a broader fraction of the compact sperm chromatin (21). Nevertheless, in our study (Figure 2), SCSA ranges from 0 to 4.45, while TUNEL ranges from 0.1 to 74.2 % and linear regression of SCSA (tDFI) on TUNEL explains 10.4 % of SCSA (tDFI) variation. Therefore, although the correlation exists, it is low and the magnitude between techniques is considerably different. SCSA data are processed for each spermatozoon with a series of cutoff values of the red/total fluorescent ratio. DFI acronym is used both to refer to the red/total fluorescent ratio of individual spermatozoa and to refer to the percentage of spermatozoa with moderate and high DFI (22). The difference in values could be due to the cutoffs used because although the SCSA test is relatively easy to follow, many factors can influence its results (23). The preparation of a standard sample, denaturation, and staining conditions and times must be strictly controlled, and the AO must be of the highest quality. Adjustment of the sperm concentration and dilution with the AO solution must be carefully performed because AO must be at equilibrium with the sperm sample (6). On the contrary, TUNEL is more complex to perform but provides precise information about the degree of sDF because fluorescence increases with the number of 3‘hydroxyl ends. The TUNEL data are only a single parameter as a ratio of total sperm to FITC-positive sperm (staining of free 3 hydroxyl ends of ss DNA).

Many papers (20, 24–26) maintain that the TUNEL test detects existing DNA strand breaks, but the SCSA detects *potential* DNA strand breaks suggesting that the acid step causes DNA strand breaks. Contrary, Evenson et al. (7) concluded that the SCSA test detects existing DNA strand breaks just as the TUNEL assay. Similarly, Gorezyeca et al. (27) showed a high correlation (*r* = 0.87, *p* < 0.05) between the two tests. However, these studies were performed with a microscope rather than adapted to flow cytometry as is the case in our results.

### Relationship Between sDF and Fertility/Prolificacy Direct Boar Effect (DBE) Score of Each Male

DBE is a very valuable score that shows information about the reproductive performance of the recorded life of a particular boar. The boar-dependent parameter DBE was compared to a punctual measurement of sDF of a particular ejaculate from each respective boar. No correlations were found between sDF from the different techniques and DBE for fertility, total born, or stillborn piglets. While DBE depends on many ejaculates that produced doses to inseminate sows during the production life of the boar, sDF was measured in a specific ejaculate extracted on a specific date.

It is known that DNA fragmentation is influenced by the seasonality and the age of the boar (27). In temperate climate countries, even when good quality semen doses are delivered from AI stations regarding sperm counts, motility, and abnormalities, results in farms are impaired in certain periods of the year, related to temperature and photoperiod (28). Despite the farm, this fact has a strong boar-related effect (29). Likewise, (30) found that the age of the boar had a significant effect on DNA stability and chromatin structure. Age of the boar at the collection and at semen production was negatively correlated with tDFI (27).

While sDF index is measured for a specific ejaculate, DBE is an intrinsic value of all the productive life of the boar. The age of the boar in the timeframe of the ejaculate extraction plus the season could influence the DNA fragmentation measurement obtained. On the contrary, the DBE depends on several reproductive outputs of all the artificial inseminations carried out with seminal doses of the boar along his lifetime. Shortly, we were comparing a punctual analysis of an ejaculate to an index that results from the reproductive outputs of several different ejaculates of the same boar along its lifetime.

### Relationship Between sDF and Characteristics of the Spermiogram

No correlations between any sDF measurements and motility were found. From many articles about numerous species using five different sDF tests, the correlations between sDF and classical semen parameters including sperm count, motility, and morphology are generally low showing that sDF is a relatively independent parameter (31). Thus, sDF is considered an independent parameter that adds different information on the quality of semen samples apart from the routine spermiogram (32).

However, a positive and low correlation was found between sDF (TUNEL) and abnormal acrosome morphology. This result could be explained because the DNA damage would be caused by an external agent such as oxidative stress (reactive oxygen species activity) damaging cell membranes, including acrosomes in addition to the DNA breaks. This hypothesis was proposed in the revision of Evenson (7). Nevertheless, in our research, the intensity of relation is very low.

The rest of the morphological abnormalities in our research were registered following a hierarchy. If a sperm cell had more than one abnormality, just the first one in the hierarchy order was registered. This procedure is not aligned with the WHO recommendations (33) and may underestimate abnormalities, as they are last in the order: acrosomal defects, abnormal heads, loose heads, abnormal tail, bent tail, and proximal and distal droplets.

**Figure 3:**
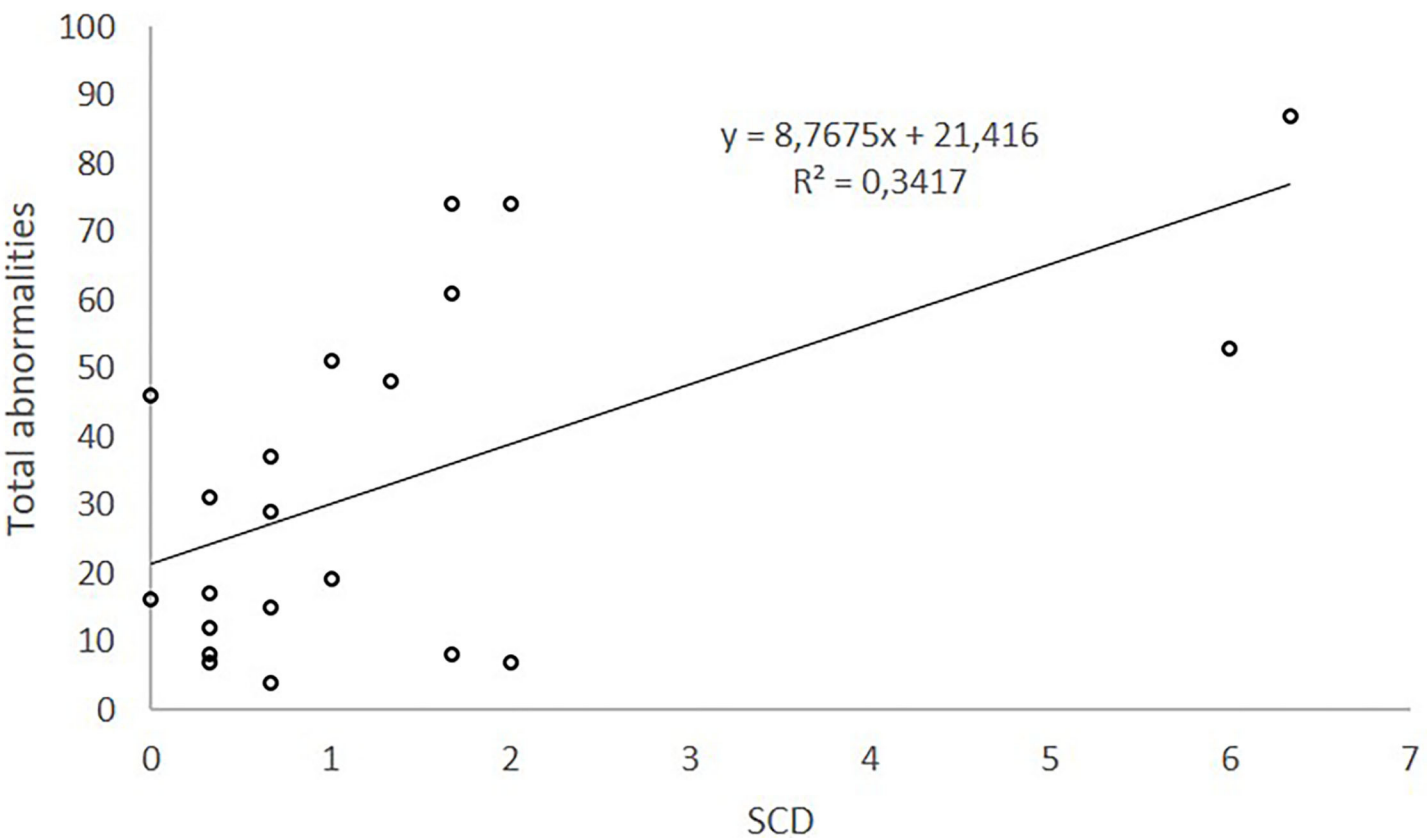
Relationship between total abnormalities and sDF measured by SCD.

On the contrary, when morphological abnormalities were globally considered (total abnormalities %), they were positively correlated with SCD which explained the 34.2% of its variability (Figure 3).

The SCD test is a simple method in kit form that measures the absence of damage rather than the damaged DNA in sperm under bright-field microscopy (7). Discrimination of sperm cells containing fragmented nuclear DNA relies on the extreme peripheral diffusion of their chromatin fragments, whereas those boar sperm nuclei without DNA fragmentation do not disperse or show very restricted spreading of DNA loops close to the flagellum (34, 35). Because the boar sperm head consists almost entirely of DNA, subtle differences in sperm head morphometry might be related to DNA status. This relationship between SCD and sperm morphological abnormalities was already described in bulls (36, 37). On the contrary, López-Fernández et al. (38), working with boar sperm, did not find significant correlations between sDF and acrosome status, frequency of distal droplets, coiled tails, and abnormal head morphology. However, the presence of proximal cytoplasmic droplets showed a significant correlation with the level of sDF observed in the ejaculated spermatozoa.

There are many factors involved in the mechanisms and causes of sDF. Intrinsic factors have been described such as oxidative stress (39), endogenous endonuclease and caspase activation (40), and alterations during meiosis or spermiogenesis (41, 42). External factors like chemicals and environmental toxicants could be involved as well (42). All these mechanisms can affect DNA integrity but also could play a role in the mechanisms of morphological abnormalities in agreement with our results.

### Relationship of sDF and Reproductive Outputs (Fertility and Prolificacy) of Sows Inseminated With the Ejaculates

High sDF has been related to reduced fertility, longer times to pregnancy, and higher spontaneous miscarriage rates in humans (15, 43). In boars, it has been described a relation between pregnancy rate and total born piglets (44). As seen in previous sections of this research and in the literature, sDF is not related to other classic sperm quality parameters but it has been proposed as a candidate to explain part of the boar-related effect of sub-fertility (7, 45).

In our study, no significant effect of the sow cycle or sDF technique was detected on fertility. This contradicts previously published reports where sDF (DFI) correlated with low semen quality or poor fertility results (4, 43).

Furthermore, no statistically significant correlations were found between total born piglets and sDF measures from the different techniques, adjusting for the sow's cycle. However, in the case of total born piglets and SCSA measure, the correlation value was close to significance pointing to a tendency for total born piglets to decrease as SCSA measure increases. This tendency would agree with results obtained by Boe-Hansen et al. (44) that studied the relationship between SCSA data from extended boar semen and field prolificacy. For Hampshire, Landrace, and Large White breeds, litter size (total born piglets) decreased by 0.5, 0.7, and 0.9 piglets per litter as % DFI values were above 2.1%. Didion et al. (4) noted the significant correlation between DFI values and an average number of piglets per litter. Since oocytes do not discriminate against sperm with damaged nuclear DNA, this DNA-damaged sperm likely fertilizes the egg, and the resulting embryos implant in the female. Those embryos fertilized with high nuclear DNA fragmented sperm may be lost later when likely needed proteins are lacking due to a break in the DNA/gene required for supplying that vital protein (7).

However, in this study, a tendency was observed but data did not show a significant correlation. This lack of statistically significant correlation could be explained because there are a lot of variables not related to boar (semen doses) affecting reproductive outputs (46). There were no samples with very high Sdf, and therefore, dramatic effect on fertility and prolificacy was not detected because small differences in sDF that might slightly influence could be obscured by the many variables that influence reproductive parameters on commercial farms. Large variation in the fertility and prolificacy results has been reported, mainly due to farm and sow-related parameters (1). *In vivo* fertility varies among farms, with boar-related parameters accounting only for 6% of total variation (29). Due to this multifactorial influence in the reproductive output, it seems difficult to find a statistically significant correlation between sperm DNA fragmentation and prolificacy through an observational study. Thus, we propose a directed study controlling all the farm and sow-related variables.

## Conclusions

There are considerable differences among the results obtained from the different techniques for measuring sDF in boars, pointing to either the DNA damage measured is different or the sensitivity of the techniques is not comparable. In any case, sDF results are not correlated with total sperm motility nor progressivity of the same ejaculate. SCD showed significant correlation with total morphological abnormalities, while TUNEL was slightly correlated with acrosome defects, meaning that the information from sDF tests is mostly independent of the classical spermiogram characteristics.

It was demonstrated that a punctual measurement of sDF in a particular ejaculate was not correlated with the reproductive score of the boar if it was yielded from its entire past productive life (DBE). On the contrary, although sDF in ejaculates does not seem a valuable prognostic tool to predict final fertility outcomes in the sows inseminated, it could predict to some extent the prolificacy results (total born piglets). Therefore, we can conclude that sDF techniques offer extra information that could be useful for the management of AI studs but the impact of sDF problems could be masked by other factors not exclusively related to the male. However, the pressure in reducing the number of spermatozoa in the commercial doses or the increment of homogeneity in sows and farms related factors could highlight the importance of sperm DNA health.

## Data Availability Statement

The raw data supporting the conclusions of this article will be made available by the authors, without undue reservation.

## Ethics Statement

Ethical review and approval was not required for the animal study because the data was extracted from animals in production without any change of housing or management.

## Author Contributions

Conceptualization and writing–original draft: RA and JM. Data curation: RA, JM, and AB. Formal analysis and writing—review and editing: RA, JM, MT, and MF. Funding acquisition: RA, JM, AB, and MF. Investigation and supervision: JM and MF. Methodology: RA, JM, AB, and MT. Project administration and resources: JM. Validation: JM, MT, and MF. Visualization: JM and MT. All authors contributed to the article and approved the submitted version.

## Funding

This research was partially supported by the University of Zaragoza: *III convocatoria de ayudas para el Desarrollo del programa de doctorados industriales/empresariales* published in the *Boletin Oficial del Estado, n*°*1*, 2^nd^ of January 2018.

## Conflict of Interest

RA and NM were employed by company Magapor S.L. JM was employed by company CiencIAnova Magapor. The remaining authors declare that the research was conducted in the absence of any commercial or financial relationships that could be construed as a potential conflict of interest.

## Publisher's Note

All claims expressed in this article are solely those of the authors and do not necessarily represent those of their affiliated organizations, or those of the publisher, the editors and the reviewers. Any product that may be evaluated in this article, or claim that may be made by its manufacturer, is not guaranteed or endorsed by the publisher.
